# Risk Stratification Using ^18^F-FDG PET/CT and Artificial Neural Networks in Head and Neck Cancer Patients Undergoing Radiotherapy

**DOI:** 10.3390/diagnostics11091581

**Published:** 2021-08-31

**Authors:** Sebastian N. Marschner, Elia Lombardo, Lena Minibek, Adrien Holzgreve, Lena Kaiser, Nathalie L. Albert, Christopher Kurz, Marco Riboldi, Richard Späth, Philipp Baumeister, Maximilian Niyazi, Claus Belka, Stefanie Corradini, Guillaume Landry, Franziska Walter

**Affiliations:** 1 Department of Radiation Oncology, University Hospital, LMU Munich, 81377 Munich, Germany; lena.minibek@med.uni-muenchen.de (L.M.); Richard.späth@med.uni-muenchen.de (R.S.); Maximilian.niyazi@med.uni-muenchen.de (M.N.); Claus.belka@med.uni-muenchen.de (C.B.); Stefanie.corradini@med.uni-muenchen.de (S.C.); Franziska.walter@med.uni-muenchen.de (F.W.); 2 Department of Medical Physics, Ludwig-Maximilians-Universität München, Am Coulombwall 1, 85748 Garching, Germany; elia.lombardo@med.uni-muenchen.de (E.L.); Christopher.kurz@med.uni-muenchen.de (C.K.); Marco.Riboldi@physik.uni-muenchen.de (M.R.); guillaume.landry@med.uni-muenchen.de (G.L.); 3 Department of Nuclear Medicine, University Hospital, LMU Munich, 81377 Munich, Germany; Adrien.holzgreve@med.uni-muenchen.de (A.H.); lena.kaiser@med.uni-muenchen.de (L.K.); Nathalie.albert@med.uni-muenchen.de (N.L.A.); 4 Department of Otorhinolaryngology, Head and Neck Surgery, University Hospital, LMU Munich, 81377 Munich, Germany; Philipp.baumeister@med.uni-muenchen.de; 5 German Cancer Consortium (DKTK), Partner Site Munich, 81377 Munich, Germany

**Keywords:** HNSCC, artificial neural network, 2-[^18^F]FDG PET/CT, UMAP, feature extraction, Harrell’s concordance index

## Abstract

This study retrospectively analyzed the performance of artificial neural networks (ANN) to predict overall survival (OS) or locoregional failure (LRF) in HNSCC patients undergoing radiotherapy, based on 2-[^18^F]FDG PET/CT and clinical covariates. We compared predictions relying on three different sets of features, extracted from 230 patients. Specifically, (i) an automated feature selection method independent of expert rating was compared with (ii) clinical variables with proven influence on OS or LRF and (iii) clinical data plus expert-selected SUV metrics. The three sets were given as input to an artificial neural network for outcome prediction, evaluated by Harrell’s concordance index (HCI) and by testing stratification capability. For OS and LRF, the best performance was achieved with expert-based PET-features (0.71 HCI) and clinical variables (0.70 HCI), respectively. For OS stratification, all three feature sets were significant, whereas for LRF only expert-based PET-features successfully classified low vs. high-risk patients. Based on 2-[^18^F]FDG PET/CT features, stratification into risk groups using ANN for OS and LRF is possible. Differences in the results for different feature sets confirm the relevance of feature selection, and the key importance of expert knowledge vs. automated selection.

## 1. Introduction

Advances in radiation oncology and medical imaging are closely linked, as attested by the widespread use of image guided radiotherapy (IGRT) and, more recently, the successful implementation of MR guided radiotherapy [1,2]. The past decade has seen strong growth in the extraction of quantitative features from medical imaging which can be used in predictive models; a practice called radiomics [3]. The application of machine learning provides the possibility of processing a high number of heterogeneous parameters obtained from clinical imaging data. Using artificial intelligence algorithms and specifically neural networks for machine learning allows for processing large amounts of data for predictive model building [4]. This development has the potential to translate into clinical practice by guiding treatment decisions and therapy planning, especially in an imaging driven field such as radiation oncology [5], where for instance several studies [6,7,8] showcased how deep learning could be used for head and neck cancer outcome prediction based on pre-treatment CTs. Imaging with PET/CT using 2-deoxy-2-[^18^F]fluoro-D-glucose (2-[^18^F]FDG) is routinely part of pretreatment workup in several tumor entities such as squamous cell carcinoma of the head and neck (HNSCC) and allows extraction of several features. It has been shown that the diagnostic value of 2-[^18^F]FDG PET/CT has an impact on therapy decisions in HNSCC [9,10,11,12]. The prognostic value of several first order PET features, such as maximum of the standardized uptake value (SUV_max_) or total lesion glycolysis (TLG) [13,14,15], has been previously studied. In PET images, volumes of interests (VOIs) can be defined semi-automatically based on the tracer-uptake, eliminating the need to manually define VOIs for radiomics evaluation, allowing for high-throughput user-independent evaluation.

Today the cornerstone of treatment decision making in radiation therapy of HNSCC is the knowledge of risk factors that are well described in the literature. These risk factors include clinical data (e.g., age, sex, smoking history), tumor classifications (e.g., TNM-Stage), and histological features (e.g., grading, HPV-status) [16,17,18]. HPV expression has been shown to be an independent prognostic factor for overall survival in patients with oropharyngeal carcinoma [17]. However, even among HPV negative tumors a wide range of different therapy responses are reported [19]. Therefore, means of further treatment stratification and personalized tailoring of therapy strategies are needed. Artificial neural networks (ANNs) [20] based on image features are a promising approach.

In this study, we investigated whether a neural network-based algorithm applied on PET features along with clinical data can provide prognostic information for head and neck cancer patients undergoing curative radiotherapy in terms of locoregional failure (LRF) and overall survival (OS). This approach is novel since it relies entirely on features extracted from semi-automatically generated PET VOIs, and is thus not relying on expert segmentation. We specifically evaluated the impact of input feature selection on the neural network’s performance.

## 2. Materials and Methods

Approval by the Institutional Ethics Committee of LMU Munich (protocol code No. 448-13 (date of approval 21 October 2013) for the retrospective data evaluation was available in the context of the clinical cooperation group (KKG) “Personalized radiotherapy for head and neck tumors”.

### 2.1. Patients

We identified patients who underwent curative therapy according to international guidelines with either complete surgery with neck dissection and adjuvant (chemo)radiotherapy (RCT) or definitive RCT for primary head and neck tumors between 06/08 and 02/20. Only patients with pre-treatment 2-[^18^F]FDG PET/CT, older than 18 years, and with a follow-up history of at least 1 year were included. Tumor stage was assessed using the UICC 7th edition classification [21]. Patient and tumor characteristics, as well as the treatment modalities, were evaluated. A complete list of the clinical features is given in Table A1 in the Appendix B.

Follow-up has been calculated from the first day of the definitive or adjuvant RCT. The events of the survival endpoints were defined as follows: overall survival (OS)—time in months from first day of radiotherapy until death, and locoregional failure (LRF)—defined as local or regional recurrence histologically proven by needle biopsy or surgery.

### 2.2. 2-[^18^F]FDG PET/CT Imaging Protocol

Whole-body PET/CT images were acquired on a Biograph mCT Flow 20-4R PET/CT scanner (Siemens, Healthcare GmbH, Erlangen, Germany) and a GE Discovery 690 PET/CT scanner (General Electric, Munich, Germany) as previously reported [22,23,24]. Patients fasted at least 4 h prior to administration of approximately 250 MBq 2-deoxy-2-[^18^F]fluoro-D-glucose (2-[^18^F]FDG), and emission scan acquisition of the immobilized head and neck, thorax, abdomen, and pelvis started approximately 60 min after intravenous tracer administration. Unless contraindicated, iodine-containing contrast medium (Ultravist 300, Bayer Vital GmbH, Leverkusen, Germany or Imeron 350, 2.5 mL/s, Bracco Imaging Deutschland GmbH, Konstanz, Germany) was administered for diagnostic computed tomography (CT) imaging (100–190 mAs, 120 kV; portal venous phase).

### 2.3. Feature Extraction

Delineation of the tumor region from which the features were extracted was performed semi-automatically using the HERMES Browser (P5, Gold, Version 4,17; HERMES Medical Solutions AB, Stockholm, Sweden) with the true attenuation corrected (AC) reconstruction. Firstly, a broad ROI was labeled by an expert physician. The SUV_max_ and SUV_peak_ representing the mean SUV of the voxels within a 1 cm^3^ cubic VOI around SUV_max_ were determined by the software. Then multiple volumes of interest (VOIs) of the area of the primary tumor covered with 25% (SUV25), 40% (SUV40), 50% (SUV50), 75% (SUV75), and 90% (SUV90) of the SUV_max_ or more were automatically delineated. SUV_min_ and SUV_mean_ were determined, and the mean tumor volume (MTV) and total lesion glycolysis (TLG) were calculated for every VOI. Additionally, SUV_mean_ liver [25], SUV_mean_ cervical spine [26], and SUV_mean_ aorta were defined via delineation and a set of ratios were calculated [27] as follows.

SUV_max_ − Ratio (SUR_max_):

SUV_max_ was divided by SUV_mean_ of liver, spine, and aorta respectively (referred to as Organ in the following formulas) to assess the ratio SUR_max_ of SUV_max_ of the tumor and the corresponding organ.

(1) $$SUR_{max}\;Organ=\frac{SUV_{max}\;Tumor}{SUV_{mean}Organ}$$

SUV_mean_ − Ratio (SUR_mean_):

Tumor SUV_mean_ values of SUV40, SUV50, SUV75, and SUV90 were divided by SUV_mean_ of liver, spine, and aorta respectively.

(2) $$SUR_{mean}Organ=\frac{SUV_{mean}\;of\;SUV40,\;SUV50,\;SUV75\;or\;SUV90}{SUV_{mean\;}Organ}$$

TLG values of SUV40, SUV50, SUV75, and SUV90 were divided by SUV_mean_ of spine, liver, and aorta respectively.

(3) $$SUR_{TLG}\;Organ=\frac{TLG\;of\;SUV40,\;50,\;75\;or\;90}{SUV_{mean\;}Organ}$$

### 2.4. Data Preprocessing

Our input covariates comprised both numerical (e.g., age, SUV values, etc.) and categorical (e.g., sex, tumor site, etc.) data. Missing observations for the numerical values (2.6% on average, range 0.4% to 7.0%, excluding all covariates where no observation was missing) were replaced with the median value for that covariate while missing observations for categorical values (2.8% on average, range 0.4% to 8.7%, excluding all covariates where no observation was missing, grading and HPV-status, see below) were replaced with the most common class for that covariate. Z-score normalization was applied to the numerical variables to have zero mean and unit variance. The standardization was fitted on the training data and then applied without changes to both training and testing numerical covariates (see Model Optimization and Details subsection for the adopted training and testing data subdivision). The categorical variables were one-hot encoded, meaning that, e.g., for the sex variable, a male was represented as a 2D-vector with a one in the first entry and a zero in the second entry and a female was represented as a 2D-vector with a zero in the first entry and a one in the second entry of the vector. For the grading and HPV-status categorical variables, the number of missing observations was particularly high, amounting to 13.9% and 41.3% of all patients. Therefore, instead of replacing missing observations with the most common category, we considered ‘unknown status’ as an extra category for the one-hot encoding procedure.

### 2.5. Feature Selection

Within this study, three different feature selection methods were used. To build the first set of input variables, an advanced dimensionality reduction algorithm called Uniform Manifold Approximation and Projection (UMAP) [28] was leveraged to automatically extract as much information as possible from all the available covariates. The UMAP algorithm constructs an abstract high-dimensional representation of the input data and then optimizes a low-dimensional representation to be as structurally similar as possible to the high-dimensional one. In other words, the UMAP algorithm can convert a high number of initial (and potentially redundant) features into a small number of new features (embedding), trying to preserve all information contained in the initial set. We fitted the UMAP algorithm on the training data and then applied it to both training and testing data to generate the embeddings. To compare the predictive performance of a model using an automatically extracted embedding of features as input, we built two additional models based on two different sets of features selected from all the available covariates by an expert radiation oncologist.

The first physician-selected feature set comprises classical clinical covariates which have been shown to be predictive in previous studies and which would be available without 2-[^18^F]FDG PET/CT [19]. We called this set of input variables Literature Only (LO) features and used it as baseline to evaluate whether additional PET features could improve the results. The selected variables are described in Table 1.

The second physician-selected feature set comprises the same LO features plus some expert-selected PET features that are easily extractable from every diagnostic PET software and with a focus on SUR_max_, SUR_mean_, and SUR_TLG_. We called this set of variables Literature and PET (LP) features. The selected PET features are listed in Table 1. While final physician feature selection is presented in the results section, this was done only once prior to model optimization.

### 2.6. Artificial Neural Network

In this work, we used Nnet-survival [29], a non-linear adaptation of the Cox proportional hazards model, to extend a binary classification ANN to a survival analysis one. Our ANN can thus incorporate censoring information and outputs survival curves/recurrence-free probability curves as a function of time for every patient.

### 2.7. Model Optimization and Details

**Figure A1:**
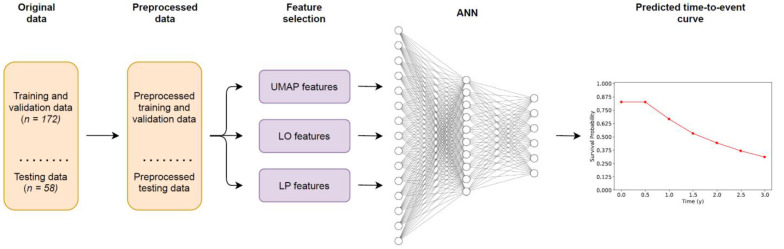
Workflow of the proposed models. The input covariates are first split randomly into training and testing data and preprocessed. Then, three different feature selection methods are used, one algorithmic (UMAP) and two based on expert knowledge (LO and LP). An ANN extended with a previously published survival model is applied on the three different embeddings to predict time-to-event curves for each patient, as shown exemplarily for one patient.

The overall workflow used in this study is shown in Figure A1. The training/validation data and the testing data were obtained by randomly taking 75% and 25% out of the entire dataset of 230 patients. This procedure was done only once, prior to all trainings. The 172 training and validation patients were used to find the best hyper-parameters of the models. Specifically, for each ANN applied to a different embedding, we performed an automatic grid search over all 18 combinations of the learning rate (1 × 10^−4^, 5 × 10^−4^ and 1 × 10^−3^), the number of hidden layers (1, 2), and the number of neurons in the hidden layer (5, 10, 15). When the ANN was applied to the expert knowledge features, the grid search was performed only once over the 18 above-mentioned combinations. On the other hand, the algorithmic UMAP dimensionality reduction has its own parameters: specifically, we decided to vary the number of nearest neighbors (5, 15, 25, 50), which controls how UMAP balances local versus global structure of the data and the number of features of the embedding (5, 15, 25, 50). Thus, when performing the grid search for the UMAP + ANN model we looked at all 18 combinations for the ANN and at all 16 combinations for the UMAP at the same time, leading to a grid search over 288 different combinations. For each of the combinations a 3-fold cross-validation was used and the hyper-parameters, which led to the best validation performance when averaged over the three sub-folds were selected for the final ANN. As we had three different feature sets as input to the ANN, three different sets of hyper-parameters were found per endpoint. Table 2 shows a summary of the best hyper-parameters, which were consequently used for the testing phase.

After the best set of hyper-parameters was found for each model, we repeated the 3-fold cross-validation once with the best set of hyper-parameters, leading to three different trained architectures per model (one for each cross-validation fold). To obtain a single testing set prediction out of the three networks, we performed model averaging, i.e., we applied all three models to the testing set and then averaged over their predictions before computing the evaluation metrics.

**Figure 1:**
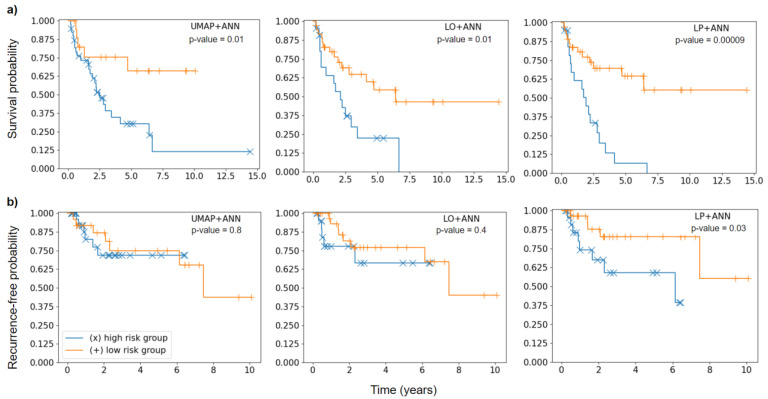
Kaplan-Meier curves of high-risk (blue) and low-risk (orange) patient groups separated according to a threshold optimized during cross-validation. Significance of difference was assessed using the log-rank test. (**a**) Kaplan-Meier plots for OS endpoint. (**b**) Kaplan-Meier plots for LRF endpoint.

All code, from data pre-processing to model building, was written in Python 3.8. The networks were optimized using the high-level API Keras 2.4.3 with Tensorflow 2.3.0 as backend. Training and testing were carried out on an NVIDIA Titan V with 12 GB of memory. We used a fixed weight decay [30] of 1 × 10^−4^ and a dropout rate [31] of 25% to avoid overfitting. The exponential linear unit [32] was used as activation function and the Adam algorithm [33] to optimize the network weights. We set the number of neurons in the output layer to six and chose a time-gap of half a year between the different output time-points. Therefore, our ANN outputs time-to-event curves for a duration of three years (see Figure 1, on the right). For all ANNs a batch size of 32 and 3000 epochs were used for training. Keras early stopping callbacks with a patience of 1000 epochs were used to terminate training if no improvement in performance was observed.

### 2.8. Statistical Analysis

The performance of the models was evaluated in two ways. First, we measured discriminative performance by using Harrell’s concordance index (HCI) [34]. HCI is commonly used in survival analysis as it quantifies for how many pairs of patients the predicted risk and the ground truth time-to-event or last follow-up time are concordant. HCI is normalized and amounts to 1.0 if all possible pairs are concordant and to 0.5 if we would assign random risks to the patients. Our model outputs time-to-event curves, so to compute HCI we have chosen the survival probability and additionally recurrence-free probability after two years as risk value. To obtain confidence intervals for the testing set HCI we used bootstrap re-sampling [34], i.e., we repeatedly took samples with replacement from the original testing set with 58 patients to generate many testing set variants. Specifically, we applied our models to 1000 bootstrap sets, therefore obtaining for each model 1000 HCIs. From these, we computed the median HCI with 83% confidence intervals (see Discussion for details on the choice of using 83% intervals).

In addition to HCI, we quantified the model’s capability of stratification into high- and low-risk testing patient groups. For this purpose, we first found a threshold during cross-validation by averaging over the risk of all patients with event, then averaging over the risk of all patients without event and finally taking the mean of these two values to set a threshold for all three cross-validation models, thus obtaining a single model averaged threshold. We then used this threshold to split the testing set into high-risk and low-risk patients and applied the log-rank test to infer whether the difference in the two groups was significant. Results with *p*-values < 0.05 were considered significant. To visualize patient stratification, Kaplan-Meier curves were used.

## 3. Results

### 3.1. Patients

We included 230 patients, with a median age of 64 (range 28–93), 167 (72.6%) male, and 63 (27.4%) female. Regarding UICC stage, 5 (2.2%) were staged UICC I, 25 (10.9%) UICC II, 45 (19.6%) UICC III, and 155 (67.4) UICC IV. A comprehensive analysis of patient characteristics and the allocation between training and testing cohort can be seen in Table A1 (Appendix B). Median follow up was 31 months (range 1–175 months), 55 patients experienced a loco-regional recurrence, and 123 patients died, leading to a mean OS of 40.5 months (range 0.3–151.1 months) and a mean LRC of 26.2 months (range 3.1–118.6 months). Forty-one patients received surgery with adjuvant RT and 32 patients received surgery with adjuvant RCT with a median overall dose of 64 Gy (range 61.4–70.0 Gy) to the tumor bed. Definitive RCT was received by 157 patients with a median overall dose to the tumor of 69.96 Gy (range 63–70.4 Gy) applied via 3D conformal or intensity-modulated radiotherapy (IMRT). Patients with extracapsular extension of the involved lymph nodes (ECE+), close or incomplete resection status additionally received chemotherapy. Subsequent chemotherapy was administered to 189 patients: Cisplatin/5-Fluorouracil (CDDP/5-FU in accordance with the ARO 96-3 Study), 5-Fluorouracil/MMC (Mitomycin C (MMC) 10 mg/m^2^ d1, d29; 5-FU 600 mg/m^2^ d1–5), Cisplatin mono (40 mg/m^2^ weekly) or Cetuximab mono (Cetuximab 250 mg/m^2^ weekly with 400 mg/m^2^ loading dose).

### 3.2. Feature Extraction

For each of the 230 patients, 102 covariates were extracted in total. Out of these, 24 were clinical variables and 78 were PET-based variables. The size of the data matrix prior to the preprocessing step therefore equals 172 × 102 for cross-validation and 58 × 102 for testing. All PET-based variables are listed in Table A2 (Appendix B).

### 3.3. Data Preprocessing

After preprocessing, the size of the data matrix was 172 × 206 for the cross-validation set and 58 × 206 for the testing set due to the one-hot encoding procedure which increased the dimension of categorical variables.

### 3.4. Feature Selection

The size of the cross-validation and testing input feature matrix after UMAP was 172 × 50 and 58 × 50 for the OS endpoint and 172 × 15 and 58 × 15 for the LRF endpoint. The final number of features (50 for OS and 15 for LRF) is a hyper-parameter of the UMAP algorithm, so it was automatically determined during optimization. For the LO set, seven clinical variables were chosen by the expert physician, that is ‘Gender’, ‘Age at diagnosis’, ‘Tumor localization’, ‘T Stage’, ‘N Stage’, ‘Tumor grading’, and ‘HPV-p16 status’. After preprocessing, this yields an input data matrix of 172 × 43 for cross-validation and 58 × 43 for testing. For the LP set, we used the LO features plus nine expert selected PET variables, which are shown in Table 1. Therefore, 16 covariates in total were used. After preprocessing, this yields an input data matrix of 172 × 52 for cross-validation and 58 × 52 for testing.

### 3.5. Model Optimization and Details

The hyper-parameters found via cross-validation for the UMAP algorithm and the ANN are shown in Table 2. A 3-fold cross-validation took on average 2 min.

### 3.6. HCI Comparisons

With an HCI of 0.71 (0.64–0.78), the best performing model for OS was the ANN applied to literature and PET (LP) features selected by an expert physician. However, it should be noted that LP + ANN was slightly, yet not significantly, better than the other two models, as the confidence intervals overlapped. For LRF, we found the literature only based ANN, with an HCI of 0.70 (0.56–0.80), to be slightly better than the LP + ANN model; the UMAP + ANN model was inferior. The differences were again not significant in terms of confidence intervals (Table 3).

### 3.7. Risk Group Stratification

For OS, all three models achieved a significant stratification (UMAP + ANN *p* = 0.01; LO + ANN *p* = 0.01) although the separation of the two groups was more evident for the LP + ANN model (*p* < 0.001). For LRF, the LP + ANN model was the only one able to significantly divide the testing patients into high-risk and low-risk groups (*p* = 0.03). The UMAP + ANN model (*p* = 0.8) and LO + ANN (*p* = 0.4) showed worse performance in stratifying patients (Figure 1).

## 4. Discussion

Our results show that risk stratification for patients undergoing curative treatment for HNSCC using an ANN is feasible. By testing three different feature selection approaches, we were able to show that adding 2-[^18^F]FDG PET/CT features enhances the performance of the stratification process, however the differences were not statistically significant.

Generally, due to the ANN’s black-box nature it is not possible to determine which covariates were used to perform the prediction, so it could not be explicitly inferred whether the additional usage of PET features is needed for the algorithm. Theoretically, an ANN based solely on clinical data could have been enough to achieve high prognosis performance on both endpoints. However, when looking at risk group stratification for OS, the use of additional PET features (LP + ANN) led to better results than without the PET data (LO + ANN). In fact, for LRF the LP + ANN model was the only one able to significantly stratify the testing patients in high-risk and low-risk groups. For OS, all three models achieved a significant stratification. However, we noted that the separation of the two groups was more evident for the LP + ANN models, making it the best model in this analysis and thus confirming the added value of the initial 2-[^18^F]FDG PET/CT. A similar result was reported in another study by Bogowicz et al. [35], where the additional information derived from 2-[^18^F]FDG PET/CT led to superior results for local tumor control modeling than standard CT.

However, this seems to be in contrast with the result that the UMAP + ANN model, to which all PET covariates were available, showed the worst performance. As visible from the results, UMAP found some relevant information from all available covariates, but this information could not be translated by the ANN into the best performing model. Similar results were described by Ger et al. when they tested radiomics features for their additional value in initial PET and CT images of HNSCC patients. They reported a worsening of the AUC by adding radiomics features to volume of the tumor alone, showing that radiomics features are not automatically associated with survival and in general that using more features as input to a predictive model does not necessarily lead to improved results [36]. A similar potential explanation could therefore be that most of the covariates we extracted have no or only poor association with the selected endpoints, making it difficult for the UMAP + ANN to find predictive patterns. On the other hand, the finding that the LP + ANN model containing some of the PET covariates performed better than the clinical data alone (LO), suggested that at least some of the PET covariates do have an impact on the endpoints. This suggests that state-of-the-art dimensionality reduction algorithms may not be sufficient if too much unselected data is given as input.

Several studies have underlined the importance of the feature selection step prior to classification [37,38]. The superior performance of the expert knowledge features based ANNs (LO + ANN and LP + ANN) compared to the automatically extracted feature-based ANN (UMAP + ANN) suggests that not only feature selection is crucial, but also that expert knowledge can play a key role in the process. Under a clinical point of view, this result is of relevance as only a small number of known covariates would have to be collected for every patient. Within this study, 83% confidence intervals have been used for HCI as it can be shown [39,40] that if two 83% confidence intervals do not overlap, then the two-corresponding means/medians differ significantly with a significance level of 0.05, which corresponds to the statistical confidence usually reported in literature.

It has been underlined [41] how standardization of the different steps of radiomics model development will play a key role for the field to move forward. Although the Image Biomarker Standardization Initiative [42] represents a fundamental step in the standardization of feature calculation, better interpretability of the extracted features and standardization of the segmentation of the VOI from which the features are calculated remain challenging. In fact, in most radiomics studies the VOI is manually delineated by an expert radiologist or radiation oncologist [43], which introduces reproducibility issues and requires additional time if the segmentation is not part of the treatment workflow.

An advantage of our approach compared to traditional radiomics is that no manual contouring of VOIs is needed. Another advantage is that by using solely semi-automatically collected features with a commercial diagnostic software, reproducibility issues are minimized. Furthermore, the fact that neither additional software for recognizing or extracting features nor time to delineate VOIs is needed, fosters use in clinical routine.

ANNs have been successfully used for binary outcome prediction of cancer in multiple studies [44,45,46,47]. However, several authors [29,48,49] have underlined the importance of incorporating censoring information in the model optimization. In fact, in a binary classification model this information is simply discarded, and each patient’s outcome is either labeled as ‘event’ or ‘no-event’. On the other hand, a survival model (or for other endpoints than OS, a so-called time-to-event model) is built not only using the information on whether an event occurred or not, but also the information on when it occurred or if follow-up was interrupted at a certain time point (i.e., right-censoring). A standard method used for survival analysis and to predict the risk of an event is the Cox proportional hazards model [48]. In this work, we used Nnet-survival [29] to extend a binary classification ANN to a time-to-event model, therefore incorporating censoring information in our model.

The main limitation of this study is that we only included patients from a single center. However, the patient cohort consists of 230 patients including UICC Stages I-IV and patients undergoing adjuvant or definitive RCT. We therefore believe the cohort is representative and well suited for training and testing an ANN. An external validation is planned in the future and might serve not only to test the model’s reliability on patients from different centers, but also to reduce the large confidence intervals which were observed when measuring the model’s performance using HCI on the bootstrapped test set (as the number of testing patients would be larger).

## 5. Conclusions

This study could demonstrate the potential of ANNs by stratifying HNSCC patients in high and low risk groups and PET-features by further enhancing the stratification performance. Since the best results were obtained by expert feature preselection, we conclude that an arbitrarily large number of different input variables does not automatically lead to the best result, even when using a state-of-the-art dimensionality reduction technique such as UMAP. Further work is needed to confirm these results with external validation and to implement models like this one in prospective trials.

## Figures and Tables

**Table 1:** Selected features for LO and LP.

	Literature Only (LO)	Literature + PET (LP)
Clinical values	Age at diagnosis	Age at diagnosis
	Gender	Gender
	T-Stage	T-Stage
	N-Stage	N-Stage
	Tumor grading	Tumor grading
	HPV-status	HPV-status
	Smoking status	Smoking status
PET values	none	SUV40max
		SUV40peak
		SUV40TLG
		SURmax Liver
		SURmax Spine
		SURmax Aorta
		SUVmean Liver
		SUVmean Spine
		SUVmean Aorta

**Table 2:** Sets of best hyper-parameters found for the different models by performing 3-fold cross validation with several different hyper-parameter combinations.

Endpoint	Model	UMAP		ANN		
		Nearest Neighbors	Number of Features	Learning Rate	Number of Hidden Layers	Neurons per Hidden Layer
OS	UMAP + ANN	5	50	1 × 10^−3^	2	10
	LO + ANN	-	-	1 × 10^−4^	2	10
	LP + ANN	-	-	1 × 10^−3^	2	5
LRF	UMAP + ANN	50	15	1 × 10^−4^	1	10
	LO + ANN	-	-	5 × 10^−4^	2	10
	LP + ANN	-	-	1 × 10^−4^	2	15

**Table 3:** Cross-validation and testing results for the different models and endpoints. HCIs shown for the cross validation are the values obtained on each of the sub-folds while for testing we showed the median HCI with confidence intervals obtained from bootstrapping the testing set 1000 times.

Endpoint	Model	3-Fold Cross-Validation HCI	Median Testing HCI (83% Confidence Interval)
OS	UMAP + ANN	0.63; 0.59; 0.64	0.64 (0.56–0.72)
	LO + ANN	0.59; 0.65; 0.66	0.67 (0.58–0.75)
	LP + ANN	0.58; 0.66; 0.59	0.71 (0.64–0.78)
LRF	UMAP + ANN	0.55; 0.76, 0.62	0.62 (0.50–0.75)
	LO + ANN	0.55; 0.59; 0.64	0.70 (0.56–0.80)
	LP + ANN	0.56; 0.55; 0.64	0.65 (0.54–0.76)

## Data Availability

The clinical data presented in this study are available in Appendix B (Table A1).

## Appendix B

**Table A1:** Clinical features.

		Training Cohort		Testing Cohort		Proportion of Training and Testing Cohort	
		Patients	Percentage [%]	Patients	Percentage [%]	Training Cohort [%]	Testing Cohort [%]
Age at Diagnosis *	<45	9	5.20	4	7.00	69.2	30.8
	45–65	90	52.00	28	49.10	76.3	23.7
	>65	74	42.80	25	43.90	74.7	25.3
Gender *	male	127	73.4	40	70.2	76.0	24.0
	female	46	26.6	17	29.8	73.0	27.0
Tumor localization	Nasopharynx	11	6.4	4	7.0	73.3	26.7
	Oropharynx	58	33.5	22	38.6	72.5	27.5
	Oral Cavity	52	30.1	21	36.8	71.2	28.8
	Hypopharynx	27	15.6	4	7.0	87.1	12.9
	Larynx	25	14.5	6	10.5	80.6	19.4
UICC Stage	I	5	2.9	0	0.0	100.0	0.0
	II	17	9.8	8	14.0	68.0	32.0
	III	35	20.2	10	17.5	77.8	22.2
	IV	116	67.1	39	68.4	74.8	25.2
T Stage *	T1	17	9.8	5	8.8	77.3	22.7
	T2	41	23.7	16	28.1	71.9	28.1
	T3	57	32.9	9	15.8	86.4	13.6
	T4	58	33.5	27	47.4	68.2	31.8
N Stage *	N0	40	23.1	10	17.5	80.0	20.0
	N1	24	13.9	12	21.1	66.7	33.3
	N2	97	56.1	30	52.6	76.4	23.6
	N3	12	6.9	5	8.8	70.6	29.4
M Stage	M0	161	93.1	51	89.5	75.9	24.1
	M1	8	4.6	4	7.0	66.7	33.3
	Mx	4	2.3	2	3.5	66.7	33.3
Resection status	R0	17	9.8	8	14.0	68.0	32.0
	R0 (CM)	15	8.7	6	10.5	71.4	28.6
	R1	14	8.1	3	5.3	82.4	17.6
	R2	5	2.9	2	3.5	71.4	28.6
	No surgery/unknwon	122	70.5	38	66.7	76.3	23.8
Lymphovascular invasion	L0	29	16.8	15	26.3	65.9	34.1
	L1	18	10.4	1	1.8	94.7	5.3
	No surgery/unknwon	126	72.8	41	71.9	75.4	24.6
Venous tumor invasion	V0	38	22.0	16	28.1	70.4	29.6
	V1	7	4.0	1	1.8	87.5	12.5
	No surgery/unknwon	128	74.0	40	70.2	76.2	23.8
Perineural invasion	Pn0	26	15.0	11	19.3	70.3	29.7
	Pn1	9	5.2	3	5.3	75.0	25.0
	No surgery/unknwon	138	79.8	43	75.4	76.2	23.8
Tumor grading *	G1	8	4.6	0	0.0	100.0	0.0
	G2	77	44.5	25	43.9	75.5	24.5
	G3	67	38.7	21	36.8	76.1	23.9
	No surgery/unknwon	21	12.1	11	19.3	65.6	34.4
Extracapsular enhancement	ECE neg	44	25.4	19	33.3	69.8	30.2
	ECE pos.	16	9.2	3	5.3	84.2	15.8
	No surgery/unknwon	113	65.3	35	61.4	76.4	23.6
HPV-P16 status *	HPV neg	73	42.2	17	29.8	81.1	18.9
	HPV pos	26	15.0	19	33.3	57.8	42.2
	unknown/ not applicable	74	42.8	21	36.8	77.9	22.1
Smoking status *	Nonsmoker	0	0.0	0	0.0	0.0	0.0
	Smoker	158	91.3	52	91.2	75.2	24.8
	unknown	15	8.7	5	8.8	75.0	25.0
Therapy regime	OP + RT	27	15.6	14	24.6	65.9	34.1
	OP + RCT	25	14.5	7	12.3	78.1	21.9
	RCT	121	69.9	36	63.2	77.1	22.9
Death	No	79	45.7	28	49.1	73.8	26.2
	Yes	94	54.3	29	50.9	76.4	23.6

Patient characteristics * Values used for LO and LP set.

**Table A2:** Collected PET values.

SUV Values Tumor		SUV Ratio (SUR)		SUV Values Organ	
SUV40	mean	SUV40	SURmax Liver *	Spine	SUVmean *
	min		SURmean Liver		SUVmin
	max *		SURTLG Liver		SUVmax
	median		SURmax Spine *		SUVmedian
	peak *		SURmean Spine		MTV
	MTV		SURTLG Spine		TLG
	TLG *		SURmax Aorta *	Aorta	SUVmean *
SUV50	mean		SURmean Aorta		SUVmin
	min		SURTLG Aorta		SUVmax
	max	SUV50	SURmax Liver		SUVmedian
	median		SURmean Liver		MTV
	peak		SURTLG Liver		TLG
	MTV		SURmax Spine	Liver	SUVmean *
	TLG		SURmean Spine		SUVmin
SUV75	mean		SURTLG Spine		SUVmax
	min		SURmax Aorta		SUVmedian
	max		SURmean Aorta		MTV
	median		SURTLG Aorta		TLG
	peak	SUV75	SURmax Liver		
	MTV		SURmean Liver		
	TLG		SURTLG Liver		
SUV90	mean		SURmax Spine		
	min		SURmean Spine		
	max		SURTLG Spine		
	median		SURmax Aorta		
	peak		SURmean Aorta		
	MTV		SURTLG Aorta		
	TLG	SUV90	SURmax Liver		
			SURmean Liver		
			SURTLG Liver		
			SURmax Spine		
			SURmean Spine		
			SURTLG Spine		
			SURmax Aorta		
			SURmean Aorta		
			SURTLG Aorta		

* Values used for LO and LP set.
